# Design thinking during a health emergency: building a national data collection and reporting system

**DOI:** 10.1186/s12889-020-10006-x

**Published:** 2020-12-09

**Authors:** Kara N. Durski, Shalini Singaravelu, Dhamari Naidoo, Mamoudou Harouna Djingarey, Ibrahima Soce Fall, Ali Ahmed Yahaya, Bruce Aylward, Michael Osterholm, Pierre Formenty

**Affiliations:** 1 World Health Organization Avenue Appia 20, 1202 Genève, Switzerland; 2 University of Minnesota School of Public Health, 420 Delaware St SE, Minneapolis, MN 55455 USA; 3 World Health Organization, Regional Office for Africa, Cité du Djoué, P.O.Box 06, Brazzaville, Republic of Congo

**Keywords:** Design thinking, Information Management System, Disease outbreaks, Data systems

## Abstract

**Background:**

Design thinking allows challenging problems to be redefined in order to identify alternative user-center strategies and solutions. To address the many challenges associated with collecting and reporting data during the 2014 Ebola outbreak in Guinea, Liberia and Sierra Leone, we used a design thinking approach to build the Global Ebola Laboratory Data collection and reporting system.

**Main text:**

We used the five-stage Design Thinking model proposed by Hasso-Plattner Institute of Design at Stanford in Guinea, Liberia and Sierra Leone. This approach offers a flexible model which focuses on empathizing, defining, ideating, prototyping, and testing. A strong focus of the methodology includes end-users’ feedback from the beginning to the end of the process. This is an iterative methodology that continues to adapt according to the needs of the system. The stages do not need to be sequential and can be run in parallel, out of order, and repeated as necessary. Design thinking was used to develop a data collection and reporting system, which contains all laboratory data from the three countries during one of the most complicated multi-country outbreaks to date. The data collection and reporting system was used to orient the response interventions at the district, national, and international levels within the three countries including generating situation reports, monitoring the epidemiological and operational situations, providing forecasts of the epidemic, and supporting Ebola-related research and the Ebola National Survivors programs within each country.

**Conclusions:**

Our study demonstrates the numerous benefits that arise when using a design thinking methodology during an outbreak to solve acute challenges within the national health information system and the authors recommend it’s use during future complex outbreaks.

## Background

The availability of timely and accurate data during a disease outbreak is critical to decision making yet challenging to obtain, as can be seen in the COVID-19 outbreak and other acute public health events. During the 2014 Ebola outbreak in Liberia, Sierra Leone, and Guinea in West Africa, challenges were faced with the collection, management, and reporting of data [1–3]. As a rapid and effective response requires the use of data to make operational and strategic decisions, the lack of regular and accurate data limited the understanding of the outbreak, including transmission dynamics and impacted overall operations, planning and allocation of resources, and support from the international community at large [3]. At the end of the outbreak, more than 250,000 samples were tested in 47 laboratories in the 3 countries and there were an estimated 28,616 suspected, probable, and confirmed cases and 11,310 deaths [4].

The multi-faceted challenges and needs associated with collecting, managing, analyzing, and reporting data in an infectious disease outbreak lends itself to applying innovative techniques and methodologies during the responses [5]. Design thinking is a process for creative problem solving and allows ill-defined or challenging problems to be reframed in a human-centric way which focuses on the end-user and allows teams to develop practical and innovative solutions for problems [6–9]. Design thinking as a concept dates to the late 1950s in the design engineering and science fields, with one of the first models created by Herbert Simon in 1969 [6]. There are different variations of the design thinking process ranging from three to seven steps; although, all are based on Simon’s model [6, 9, 10]. Over the last few decades, the methodology has been used in numerous fields including business, education, computer science, healthcare and public health management and policy and can address a wide range of problems [6–8]. In the 1970’s, Victor Papanek, a pioneer in design thinking, collaborated with World Health Organization experts to create a low-tech malnutrition arm band for children [6, 11]. More recently, human centered design and design thinking was used to integrate tuberculosis and human immunodeficiency virus care in Kenya [12, 13], for asthma self-management in Scotland [12, 14], for dementia patients in the UK [12, 15], and for designing a backpack for school-aged children in Iran [12, 16]. It was so also used to design a surveillance and outbreak response management system for Nigeria post-Ebola outbreak [12, 17].

To address the challenges associated with collecting and reporting data during the 2014 Ebola outbreak in Guinea, Liberia and Sierra Leone, we applied a design thinking approach to build the Global Ebola Laboratory Data Collection and Reporting System [3]. We aim to demonstrate how design thinking can be used during a complex emerging pathogen outbreaks to solve acute and long-term challenges within the health information system.

## Main text

### Design thinking methodology

In building the data collection and reporting system [3], we used the five-stage Design Thinking model proposed by Hasso-Plattner Institute of Design at Stanford [9, 10]. Prior to implementing the Design Thinking methodology, we also assessed the potential usefulness of The Cynefin Framework and the Eight Disciplines Problem Solving Process to understand which methodology would be most effective and efficient in an outbreak setting to design the data collection and reporting system [18, 19]. Design Thinking was chosen as it has documented use-cases in healthcare and public health, supports rapid prototyping, is non-linear, has a low-cost of implementation, and has a low barrier to entry requiring minimal training. Additionally, it is a collaborative methodology, which is particularly important during outbreaks and health emergencies when health systems are being pushed to their limits. This approach offers a flexible model which focuses on empathizing, defining, ideating, prototyping, and testing. Engaging end-users throughout the design thinking process is paramount to ensuring that solutions are developed to meet user needs. This is an iterative methodology that continues to adapt according to the needs of the system. Therefore, many of the stages do not need to be sequential and can be run in parallel, out of order and repeated as necessary [9, 10].

### Empathizing

The design thinking process began simultaneously in Guinea, Liberia and Sierra Leone with observing, engaging, and empathizing with the current situation. This step allows for the removal of personal assumptions with a view of observing the problem through the end-user’s perspective [9, 10]. To do this, we met with Senior Leadership within the Ministries of Health and the Incident Managers to understand the challenges as it relates to the obtaining accurate and timely information and to obtain a landscape of all of the players whom would need to be involved. We then reviewed Ministry of Health and WHO daily situation reports (a document which details the number of confirmed, suspected and probable cases and deaths, and highlights the operational challenges). Next, we worked in the three countries alongside end-users and stakeholders who were collecting and analyzing the data as well as using the reports for decision making. Multiple one-on-one interviews and small workshops were carried out in Sierra Leone, Liberia, and Guinea in person and remotely via teleconference. Through these meetings, we were able to gain situational awareness and understand the unique needs that each stakeholder had in relation to data collection, management, reporting and decision making. Questions focused on understanding the current workflow, identifying bottlenecks, and the diverse end-user roles and responsibilities that the data collection and reporting system needed to support. Additional questions focused on understanding the short-term and long-term needs of the data. This information was written up after each interview and workshop and compiled into a central excel document for later review and prioritization. The key end-users and stakeholders were data collection officers, epidemiologists, information technology staff, data managers, laboratory personnel, technical experts, and senior leadership from the Sierra Leone, Liberia, and Guinea Ministries of Health.

### Defining & ideating

**Fig. 1:**
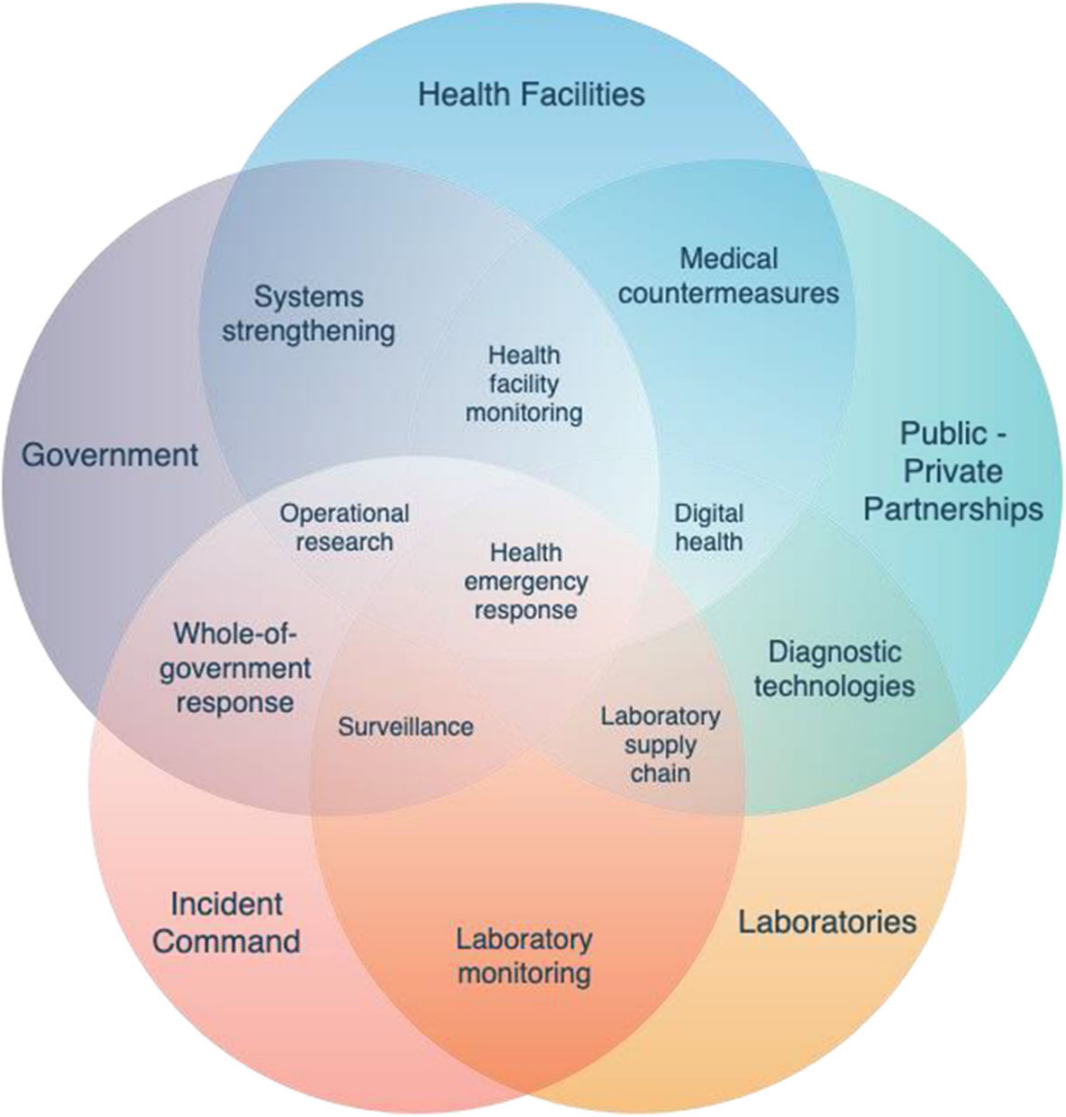
Defining the needs

Next, the end-user and stakeholder needs were defined (Fig. 1) and the problems were clearly identified and articulated. Through multiple brainstorming sessions, ideas were generated to address the list of needs and challenges of the end users and stakeholders. The ideas focused on features, functions and design characteristics essential to improving the data collection and reporting process and ranged from simple adjustments to the creation of complex systems. Prioritization of the ideas was based on speed, feasibility and flexibility due to the time constraints necessary to develop and roll-out the system.

### Prototyping

With the information obtained during the empathizing, defining and ideating stages, it was possible to view the problem from alternative ways and to design new and appropriate solutions [9, 10]. During the prototyping phase, we worked closely with IT system engineers and computer programmers to design a solution that would fit the needs of the various end-users and stakeholders according to the required list of features and functions. The list of features included searchability, standardization of data, real-time access to data, data management including cleaning and validation capabilities, and visualization of data. The list of system capabilities included data ownership and access, security, off-line use, usability with limited internet connection, versatility of languages, flexibility and adaptability to various types of users.

### Testing and redesign

**Fig. 2:**
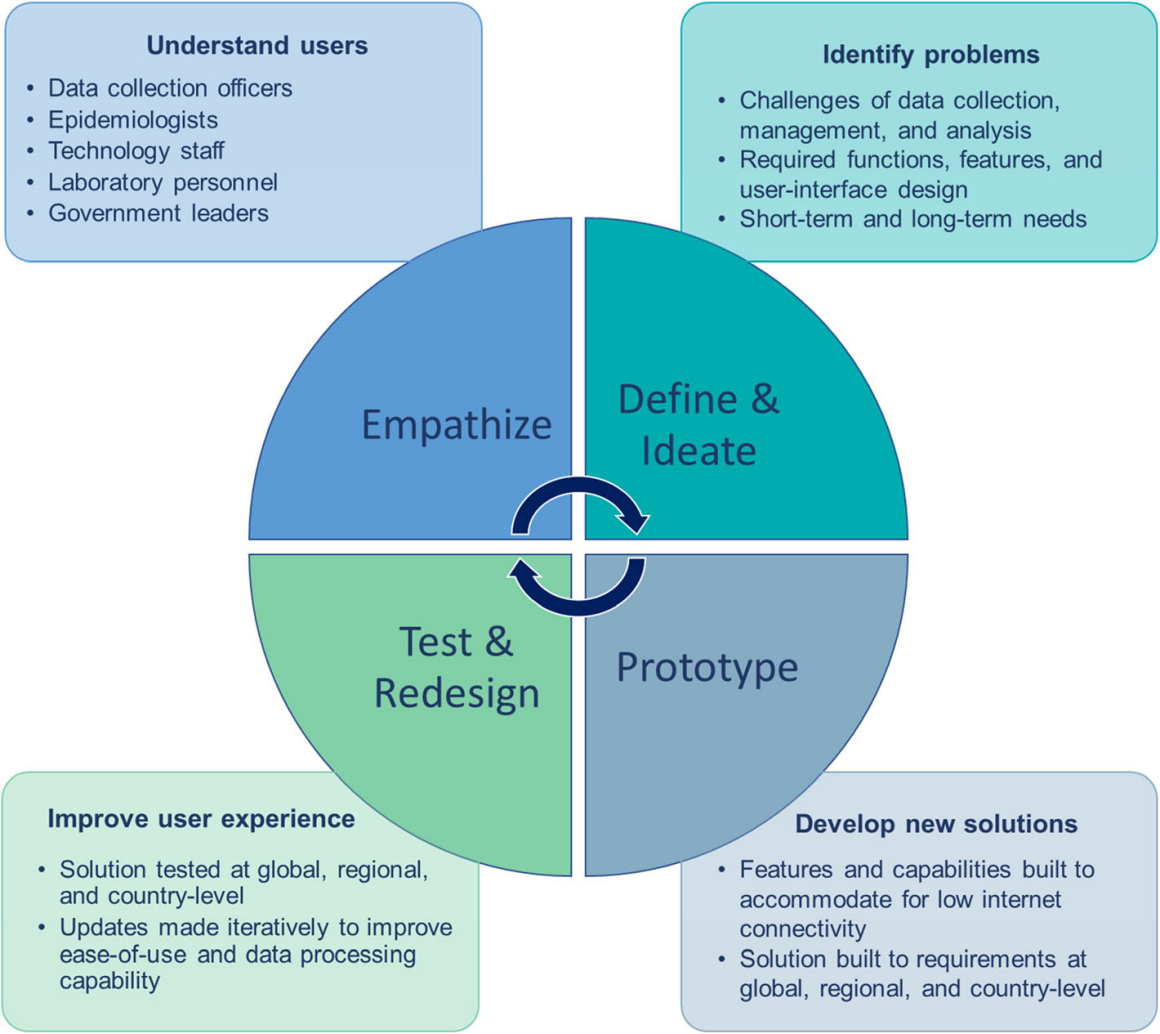
Process of implementing design thinking methodology

A prototype of the data collection and reporting system was developed over the course of 2 months. During prototyping, the focus was on identifying the best possible solutions to address the problems and requirements identified in the earlier phases [9, 10]. Lastly, the system was tested internally by the Information Technology team and subsequently rolled-out to Guinea, Liberia and Sierra Leone. Once the system was implemented, the team had regular weekly calls to discuss operational challenges and to make necessary adjustments based on the specific needs of each country. This was an iterative process with alterations and refinements being made to the system after receiving valuable feedback from end-users [9, 10]. The system took nearly 1.5 years of iterations until it was maximized to its full potential. (Fig. 2).

### Findings

From March 2014 through August 2016, the results of 256,343 specimens tested for Ebola virus disease in 47 laboratories across Guinea, Liberia and Sierra Leone were captured in the Global Ebola Laboratory database [3]. The value of the database was far reaching. It was used to orient the response at the district, national, and international levels within the three countries including generating situation reports, monitoring the epidemiological and operational situation, and providing forecasts of the epidemic [3]. It was also used to support additional Ebola-related public health interventions including the Ebola RNA persistence in semen of Ebola virus disease survivors report [20] and the Ebola National Survivors programs within each country. Further, the platform in which the Ebola data collection and reporting system was built on was adapted through end-user feedback, testing, and technology upgrades to support the 2016 Yellow Fever outbreak in Democratic Republic of Congo and Angola.

## Conclusions

Using a design thinking methodology during an outbreak allowed for buy-in and end-user expertise to drive the initial design of the system; which allowed for implementation during an outbreak. As requirements were defined by end-users for all stages of the data collection, management, analysis, and reporting phases, the first prototype that was rolled-out was immediately useable by end-users and subsequent modifications were enhancements instead of re-designs. Further, as there was buy-in from end-users, stakeholders, and leadership, it was feasible to roll-out the system simultaneously in all three countries. With active end-user feedback, it was possible to make small incremental changes over time which upgraded the system to enhance usability without impacting functionality. Small incremental changes were used instead of large system overhauls in order to not impact daily entering and reporting of the data and to minimize the amount of ancillary training that was required. Further, the on-going engagement required from the design thinking methodology resulted in strengthened communication and collaboration between and within end-users and stakeholders for harmonized strategic interventions.

Limitations of the design thinking approach for this context were threefold. Firstly, co-creating a solution with end users required sufficient contribution of their time and participation in the design process. The authors recommend that teams looking to implement design thinking during an outbreak remain cognizant of this and find agile ways of working to accommodate for valuable time that must be spent on critical response operations. A second limitation is that the focus on user centricity, especially when working “bottom-up” to create a solution, may favour certain user personas over others in the solution design. To accommodate for this, the authors worked iteratively to gather feedback from users at the country, regional, and global levels and to align needs when developing the data system design roadmap. The authors also recommend the use of project management methods alongside design thinking to manage the scope, timing, and release of data system enhancements. Lastly, critiques of design thinking in the literature note that unlike other design approaches, design thinking is poorly defined, less grounded in theory, and may narrow the potential for innovation [21]. While, the flexibility of this methodology was advantageous for the authors during an outbreak, it may not be as useful for other settings.

Cori, et al. outlined three components they found necessary in order to have useable data for analysis and decision making: 1) collecting relevant data, 2) optimizing data quality, and 3) data availability [22]. Based on challenges described in this manuscript along with Durski, et al. *Development, Use, and Impact of a Global Laboratory Database During the 2014 Ebola Outbreak in West Africa* [3], the authors agree with the recommendation from Cori, et al. and encourage this framework for future data system development. Further, in order to use design thinking to strengthen health information systems and to increase the likelihood of the output being adopted, we identified five conditions that should also be present:

i) Leadership should be involved from the beginning and have the interest and capacity to allocate resources and time accordingly. The senior leadership within the Ministry of Health and Incident Command Structure in Guinea, Liberia and Sierra Leone all supported the development of a data collection and reporting system and allocated staff accordingly to work with us in the development of the system,
ii) There needs to be an environment in which change is required. This was evident by the acute challenges associated with the outbreak and the need for and lack of timely and accurate data to inform decisions,
iii) Strong engagement with end-users and all stakeholders is necessary. Even though there were over 60 stakeholders across three countries in multiple languages, following design thinking principles allowed for a wide-range of requirements from data collectors to analysts to decision makers to be collected, analyzed, and incorporated into the final system,
iv) Strong coordination and facilitation are important. Ensuring that all stakeholders and end-users contributed to all phases ensures short-term and long-term buy-in of the system.
v) Designing and developing a system which is multi-purpose and addresses complex challenges requires everyone involved in the process to be flexible and have a growth mindset.

The inherently chaotic nature of an outbreak poses a unique set of limitations when implementing response strategies and operational research. As a result of the time pressures, the meetings with stakeholders and end-users were conducted in an ad-hoc manner by country instead of in a large, collaborative group setting by bringing together stakeholders across the region. Additionally, this was complicated due to the French and English language requirements and poor flight and transportation connectivity between Guinea, Liberia and Sierra Leone at this time of the outbreak. Additional experts in design, user experience, and data science fields could have been included in the design thinking phase. While the authors attempted to include these specialities, it was not possible due to the tight timelines of the outbreak and limited resources allocated to the creation of the data collection and reporting system. These experts could have provided unique and complementary perspectives during the design phase when working side by side with end-users. Despite numerous challenges, the design thinking methodology was paramount in developing a data collection and reporting system during one of the most complicated outbreaks to date [3]. The authors suggest the continued testing and use of design thinking to solve health system related challenges that arise during disease outbreaks and health emergencies, particularly those which rely heavily on end-users to be successful and sustainable.

## Data Availability

Data sharing is not applicable to this article as no datasets were generated or analysed during the current study.
